# HIV-1 Tat protein enhances Microtubule polymerization

**DOI:** 10.1186/1742-4690-2-5

**Published:** 2005-02-03

**Authors:** Jean de Mareuil, Manon Carre, Pascale Barbier, Grant R Campbell, Sophie Lancelot, Sandrine Opi, Didier Esquieu, Jennifer D Watkins, Charles Prevot, Diane Braguer, Vincent Peyrot, Erwann P Loret

**Affiliations:** 1 UMR Univ. Med./CNRS FRE 2737, Faculté de Pharmacie, Université de la Méditerranée, 27 Bd Jean Moulin, 13385 Marseille, France

## Abstract

**Background:**

HIV infection and progression to AIDS is characterized by the depletion of T cells, which could be due, in part, to apoptosis mediated by the extra-cellular HIV-encoded Tat protein as a consequence of Tat binding to tubulin. Microtubules are tubulin polymers that are essential for cell structure and division. Molecules that target microtubules induce apoptosis and are potent anti-cancer drugs. We studied the effect on tubulin polymerization of three Tat variants: Tat HxB2 and Tat Eli from patients who are rapid progressors (RP) and Tat Oyi from highly exposed but persistently seronegative (HEPS) patients. We compared the effect on tubulin polymerization of these Tat variants and peptides corresponding to different parts of the Tat sequence, with paclitaxel, an anti-cancer drug that targets microtubules.

**Results:**

We show that Tat, and specifically, residues 38–72, directly enhance tubulin polymerization. We demonstrate that Tat could also directly trigger the mitochondrial pathway to induce T cell apoptosis, as shown in vitro by the release of cytochrome c from isolated mitochondria.

**Conclusions:**

These results show that Tat directly acts on microtubule polymerization and provide insights into the mechanism of T cell apoptosis mediated by extra-cellular Tat.

## Introduction

AIDS is due to human immunodeficiency virus (HIV-1) infection of CD4 T cells and is characterized by the cell death of HIV-infected lymphocytes and uninfected bystander cells [1-4]. Recent discoveries have shown that this could be related to the extra-cellular effects of Tat, which is a toxic protein secreted early by HIV-infected cells [5,6]. Tat was first identified as a regulatory protein essential in the HIV viral cycle due to its ability to dramatically increase HIV gene expression [7]. The size of Tat varies and exists in forms between 86 and 101 residues. Yet 101 residues now appear to be the dominant size in the field [6]. Interest in this protein was raised with the discovery that Tat was secreted from HIV-infected cells and that it could have extra-cellular functions related to AIDS pathogenesis such as Kaposi's sarcoma [8]. The extra-cellular roles of Tat are suspected to be the major reason for the maintenance of HIV-infected cells (or reservoir cells) [5], and could explain the failure of current antiviral therapies to eradicate HIV [9]. Tat induces apoptosis in different cell lines such as macrophages and cytotoxic T-lymphocytes (CTL) [10], which are essential for the cellular response of the immune system to eliminate virus-infected cells [6]. Different mechanisms by which Tat induces apoptosis in T cells were proposed: (i) The up-regulation of Fas ligand [10]; (ii) The up or down regulation of cellular genes encoding for cytokines [11], for cell survival factors such as Bcl-2 [12-14], for superoxide-dismutase [15], and for p53 [16]; (iii) The inhibition of the expression of manganese-dependant superoxide dismutase [17]; (iv) The activation of cyclin dependant kinases [18].

Another mechanism was proposed that involves microtubules to induce Tat-mediated apoptosis [19]. We showed recently, using two short Tat variants of 86 residues, that Tat is able to form a complex with tubulin [20]. Microtubules are tubulin polymers necessary for the change and preservation of cellular morphology, intracellular organelle distribution, chromosome migration during mitosis, cell differentiation, as well as intracellular transport and signalization [21]. Microtubule damaging agents (MDAs) are classified in microtubule-stabilizing agents such as Taxanes and microtubule depolymerizing agents such as *Vinca*.alkaloids. The MDAs inhibit microtubule dynamics in living cells and lead to apoptosis after cell cycle disturbance [21-24]. They activate the intrinsic mitochondrial apoptotic pathway as shown by the mitochondrial membrane potential collapse and the opening of the permeability transition pore [25,26]. The subsequent release of pro-apoptotic factors, such as cytochrome *c*, leads to the caspase cascade activation and thus to apoptosis [27]. Moreover, MDAs can also directly affect mitochondria, as shown *in vitro *by the release of cytochrome *c *from isolated mitochondria [28].

The aim of this study was to evaluate the consequence of Tat binding to microtubules and the correlation with the mitochondria-mediated T cell apoptosis. We used three Tat variants with sequences from 99 to 101 that have the size of the main Tat variants found in the field [6]. The HIV-1 HxB2 and HIV-1 Eli are representative of rapid progressor (RP) patients in respectively Euro-American strains [29] and African strains [30]. HIV-1 Oyi strain was isolated from a highly exposed persistently sero-negative (HEPS) individual during an epidemiological study in Gabon [31]. In order to identify the regions of Tat essential for the interaction with microtubules, we used peptides corresponding to different part of the Tat sequence. We compared the effect of these Tat variants and Tat peptides with paclitaxel, a well-known anti-cancer drug agent that strongly increases tubulin polymerization and stabilizes microtubules [32].

## Results

### Tat acts on tubulin *in vitro*

**Figure 1:**
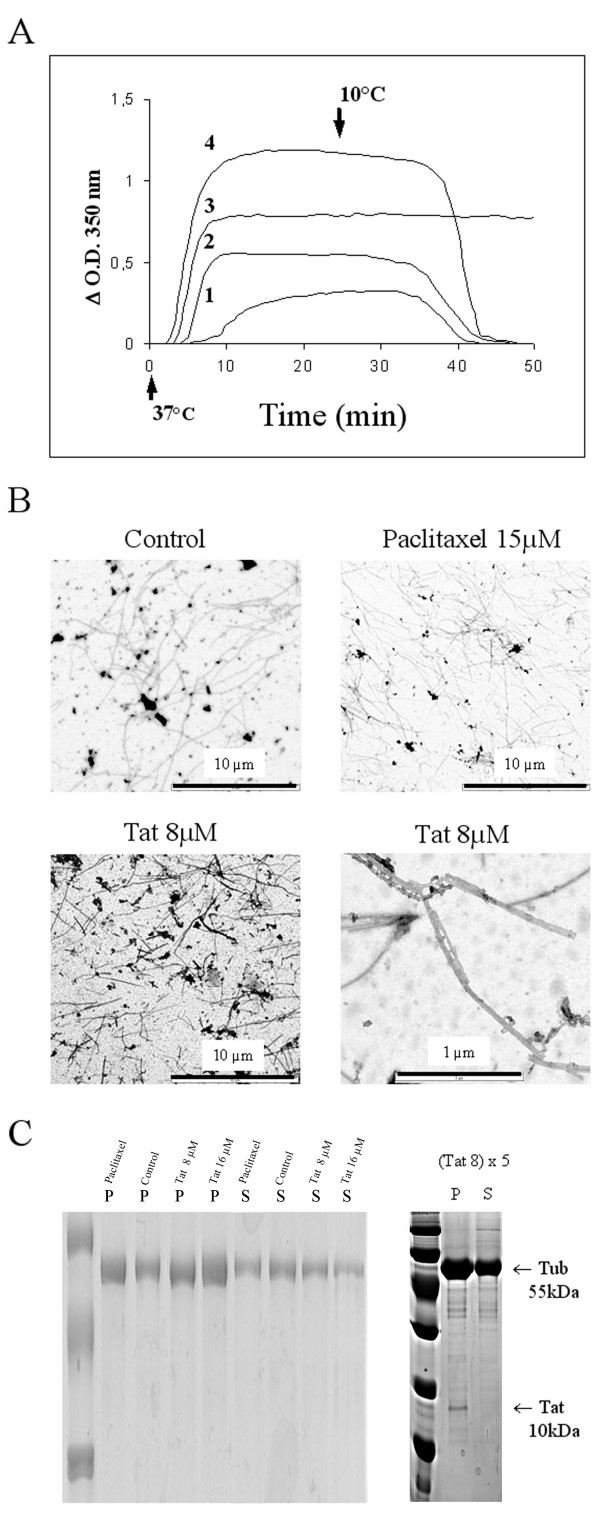
Turbidity time course of the in vitro microtubule assembly. (**A**) Tubulin in presence of various ligands. Samples with 15 μM tubulin are maintained at 4°C. After addition of 8 μM Tat (line 4), 4 μM Tat (line 2) from HIV-1 HxB2 strain or 15 μM paclitaxel (line 3), the assembly reaction was started by warming the samples at 37°C (time 0, arrow) and compared to tubulin alone (line1). The ΔOD350 nm is measured every 30 sec. After 30 min the temperature was lowered to 10°C (arrow). (**B**) Electron microscopy of microtubules formed in the presence of Tat. Aliquot from samples reaching the ΔDO350 nm plateau at 37°C were adsorbed on coated Formvar films on copper grids. Electron micrographs of microtubules formed without Tat (Control) or with indicated concentrations of Tat and paclitaxel are presented with 4000-fold magnification. Microtubules formed with Tat at 8 μM are also presented at 40000-fold magnification. (**C**) Production of microtubules in the presence of Tat. Samples of 15 μM tubulin alone (Control) and with 8 μM Tat (Tat 8), 16 μM Tat (Tat 16) or 15 μM paclitaxel (Paclitaxel) at the time they reach the plateau at 37°C were ultracentrifuged and supernatant (S) and pellets (P) were analyzed on SDS-PAGE. (Tat 8) × 5 indicates a fivefold increase in the quantity of the sample loaded. The mass of tubulin (Tub 55 kDa) and Tat (Tat 10 kDa) are indicated.

The minimal concentration of tubulin (Cr) necessary to obtain tubulin polymerization without drug was 7 μM in our buffer conditions [33]. We measured the polymerization effect of various concentrations of Tat and paclitaxel with 15 μM tubulin. We did observe the enhancement of tubulin into microtubules in the presence of Tat and paclitaxel (Fig. 1A). In comparison with tubulin alone (line 1), the rate of assembly as well as the final extent of assembly was enhanced by Tat. Furthermore, the lag time to start polymerization is shorter with Tat (lines 2 and 4) as compared with tubulin alone (line 1). With 4 μM (line 2) and 8 μM Tat (line 4), turbidity reached a plateau at 0.55 and 1.20 respectively, which corresponds to a 1.7 and 3.7 fold increase compared with the control plateau at 0.32 obtained with tubulin alone (line 1) (Fig. 1A). With higher concentrations of Tat (16 μM and 20 μM) the turbidity plateaus were superior to 5. The lowest effective concentration of Tat that induced an increase in polymerization was 0.5 μM (data not shown).

When the temperature of the samples was decreased to 10°C, we observed a complete depolymerization for tubulin alone (line 1) and tubulin with 4 μM and 8 μM Tat (lines 2 and 4 respectively). Even at the highest concentrations of Tat (16 μM and 20 μM), at 10°C the turbidity decreased to the original values (data not shown). By contrast, in the same conditions, we did not observe any depolymerization in the presence of paclitaxel (line 3). However, full depolymerization in the presence of paclitaxel was obtained with a longer incubation at 4°C (data not shown). The reversibility of following an incubation at 10°C or 4°C strongly suggests that turbidity enhancement is not related to precipitation or aggregation but to microtubule formation.

We also observed that when Tat concentration is increased to stoichiometric quantities with tubulin, the turbidity values increased dramatically to an absorbance greater than 2.

These values are superior to the absorbance obtained in the same conditions using the stoichiometric quantity (15 μM) of paclitaxel, which induces effective tubulin polymerization. Turbidity is known to be a function of the total weight concentration of scattering particles only when the particles have small diameters compared with the wavelengths of the incident light [34,35]. Thus, it seemed Tat would be able to act quantitatively and qualitatively on tubulin polymerization. In order to address these possibilities, our first control was to verify the shape of microtubules formed in the presence of Tat. Examination with electron microscopy confirmed the formation of microtubules in the presence of Tat, that were similar in shape to the controls without Tat (Fig. 1B). However, microtubules in the presence of Tat and paclitaxel were slightly packed and shorter than non-treated microtubules. These phenomena were in part responsible for the variation in turbidity. To determine whether a part of the enhancement of turbidity was due to an increase in tubulin polymerization, we did evaluate the amount of polymerized tubulin on gel after ultra-centrifugation. Results in figure 1C show that with Tat or paclitaxel the band intensity of pellets (P) are enhanced and the band intensity of supernatants (S) are decreased compared with the control tubulin alone. Increasing the concentration of Tat to 8 μM (Tat 8) and 16 μM (Tat 16) indeed enhanced the proportion of tubulin in the pellets (P) and decreased the proportion of tubulin in the supernatant (S) (Fig. 1C). Thus, although the enhanced turbidity is due in part to microtubule aggregation, these results show that the increase expansion in the turbidity plateau is also due to an increasing amount of microtubules. Interestingly, analysis of the samples by gel electrophoresis, using a five-fold amount of the tubulin samples loaded on gels showed that Tat was only detectable in the pellet fraction (Fig. 1C). To eliminate non-specific binding, we used a glycerol cushion during the ultra-centrifugation step [33]. This confirmed that Tat was associated with the microtubules.

We evaluated the ability of different Tat variants to modify tubulin polymerization. In our conditions, when 15 μM of tubulin are incubated at 37°C with 8 μM Tat HxB2, Eli or Oyi, the turbidity plateau values at 350 nm are 1.01, 1.61 and 0.80 respectively (Table 1). These values are higher than the turbidity value of 0.30 obtained with 15 μM tubulin alone (Table 1). Thus Tat from different HIV subtypes are able to enhance tubulin polymerization. However, Tat Eli and Tat HxB2 are more effective in this enhancement than Tat Oyi (Table 1, column O.D. ratio).

**Table 1:** Enhancement of tubulin polymerization by different Tat and derived peptides

Compounds	Concentration	ΔOD_350 nm_	ΔOD ratio
none		0.31	1
Tat HxB2	8 μM	1.01	3.31
Tat OYI	8 μM	0.80	2.63
Tat ELI	8 μM	1.61	5.27
Paclitaxel	15 μM	0.55	1.79
Pep38–72 OYI	8 μM	0.78	2.56
Pep38–72 ELI	8 μM	1.59	5.20
Pep73–101 OYI	8 μM	0.31	1.01
Pep73–99 ELI	8 μM	0.30	1

15 μM tubulin is incubated at 37°C in buffer, the increase of ΔOD_350 nm _is followed and the value of the plateau is indicated in the column ΔOD_350 nm_. Lane 1 is the control with tubulin alone (none), lanes 2–4 are Tat variants, lane 5 is the paclitaxel control, lanes 6–9 are the Tat peptides. The ΔOD_350 nm _ratio is the value that the ΔOD_350 nm _plateau reaches in presence of the indicated product divided by the value of the ΔOD_350 nm _plateau reached with tubulin alone.

**Figure 2:**
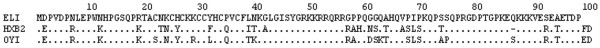
Sequences of HIV-1 Tat strains. These three Tat variants and the peptides were obtained by solid phase synthesis with a procedure previously described [43, 50]. Sequences correspond to the viral strains HIV-1 Eli [30], HIV-1 HxB2 [29] and HIV-1 Oyi [30].

To identify the sequence(s) region(s) implicated in tubulin, peptides overlapping the sequence of Tat HxB2 and Tat Oyi were synthesized. The sequences of peptides 38–72 and 73–99 derived from both Eli and Oyi are indicated in figure 2. Using 8 μM of peptide 38–72 from Eli (line Pep38–72 ELI) and Oyi (line Pep38–72 OYI) in the presence of 15 μM tubulin, we obtained a turbidity plateau value of 1.59 for Eli and 0.78 for Oyi which correspond to an increased ratio of 5.20 and 2.56 (Table 1). These effects were comparable with the effect of the entire parental Tat proteins. Turbidity with the peptide 73–99 derived from Eli (Pep73–99 ELI) and Oyi (Pep73–101 OYI) were at the same level as the control, indicating that they were not active in tubulin polymerization (last lines, Table 1).

### Tat-mediated apoptosis in T-cells correlates with the effect on microtubules

**Figure 3:**
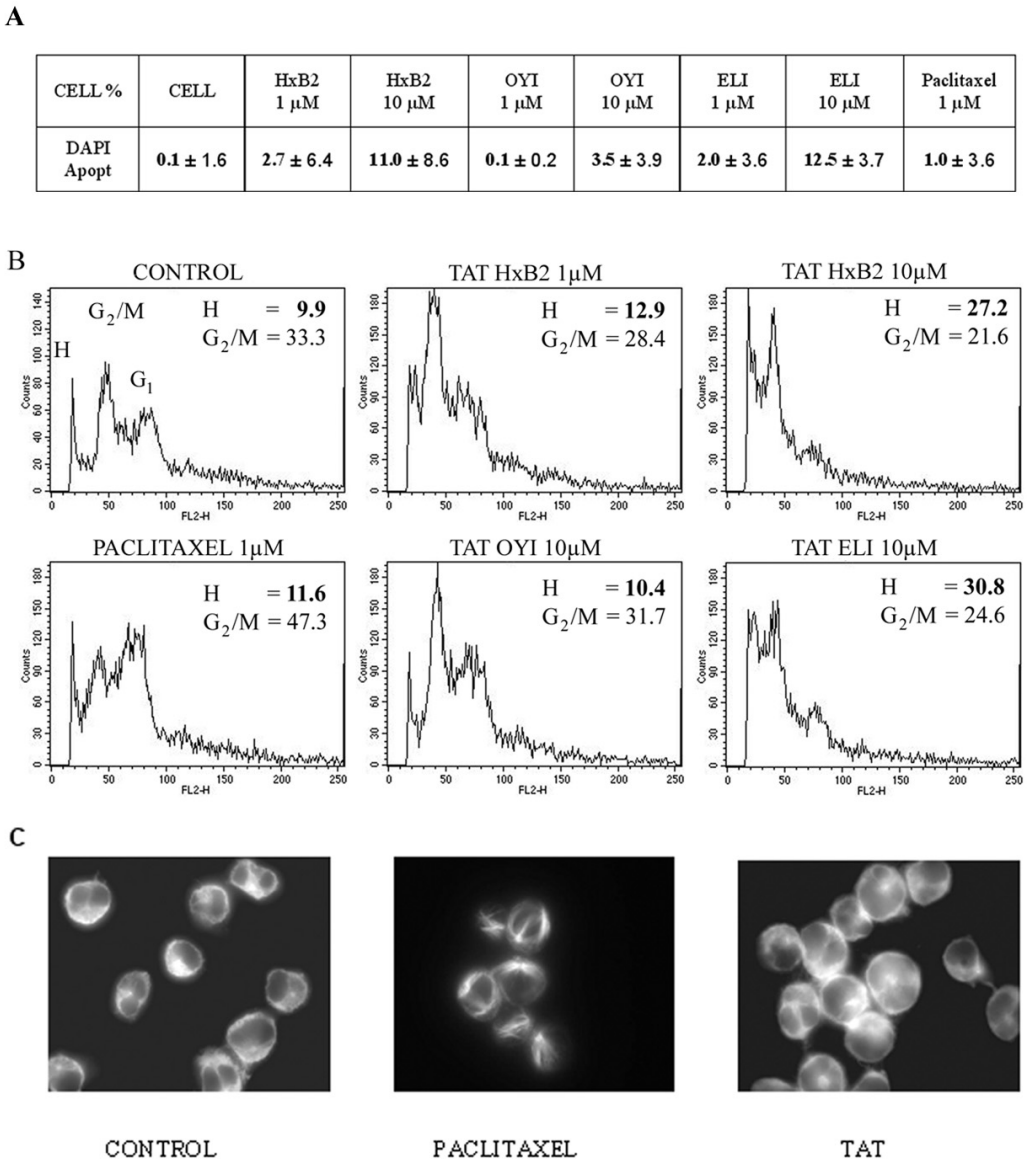
Effect of different Tat variants on cell cycle progression, apoptosis and microtubule network in lymphocytes. (**A**) Table showing data from fluorescence microscopy after 20 hours treatment with indicated concentration of Tat or paclitaxel. Percentage of apoptotic cells are determined after DAPI staining and the differences in apoptosis between treated and untreated cells used as control obtained in three independent experiments are presented. (**B**) Jurkat cells were treated with indicated concentrations of paclitaxel and various Tat or were untreated (control). After 20 hours, cells were stained with PI and analysed by flow cytometry. Percentage of cells in G2/M (G2 / M) or apoptotic cells (H) with hypodiploid DNA are indicated in the upper corner of each cell (**C**) Jurkat cells were treated with 10 μM Tat Eli or 1 μM paclitaxel and processed for immunofluorescence labeling with anti-alpha tubulin antibody as described in materials and methods. Control corresponds to untreated cells.

Drugs that are able to bind to tubulin and/or microtubules are known to affect the microtubule network organization and block the cell cycle in mitosis, leading to cell death. We first evaluated the toxicity of the different Tat variants on Jurkat lymphocyte cells, using 4'6-diamidino-2-phenylindole (DAPI) staining. This method allows the quantification of apoptotic cells by the observation of the characteristic nuclear fragmentation. After 20 hours treatment, the percentage of apoptotic cells in the presence of 10 μM Tat HxB2 (11 %) and 10 μM Tat Eli (12.5 %) were higher than those obtained with 10 μM Tat Oyi (3.5 %) (Fig. 3A). These differences in apoptosis were significant (*p *= 0.037) using one-sided ANOVA. The percentages of apoptotic cells in the presence of 1 μM Tat Oyi were similar to the control cells (0.1 %) (Fig. 3). The percentages of apoptotic cells in the presence of 1 μM Tat HxB2 (2.7 %), 1 μM Tat Eli (2 %) and 10 μM Tat Oyi (3.5 %) was weak and comparable to the basal level of the non-treated control cells (Fig. 3A). Thus, at 10 μM Tat HxB2 and Tat Eli induced apoptosis inversely to 10 μM Tat Oyi. These results indicate that Tat-mediated apoptosis in T-cells could be correlate with the effect on microtubules since the differences in toxicity between the different Tat variants correspond to the same differences in tubulin polymerization. These differences were also observed using trypan blue exclusion (data not shown). Tat toxicity was then confirmed by flow cytometry analysis after propidium iodide (Pi) staining. Figure 3B shows that 1 μM and 10 μM Tat HxB2 induces 13% and 27% apoptosis respectively, revealed by the proportion of cells with hypodiploid DNA content (Fig. 3B, H values). A high proportion of apoptotic cells (31 %) was also obtained with 10 μM of Tat Eli. Furthermore, the percentage of hypodiploid cells obtained with 10 μM Tat Oyi is low (10.4 %) and is the same as the control without Tat (9.9 %).

### Tat does not affect microtubule network organization nor cell cycle progression

Paclitaxel stabilizes the microtubule network by inducing the formation of pseudoasters in mitotic cells and the formation of bundles, structures that correspond to strongly associated microtubules, in interphasic cells [21]. Considering that Tat increased microtubule polymerization, we investigated if Tat was able to induce bundle formation or another disturbance in the microtubule network organization in the treated cells. The effects of Tat and paclitaxel cytotoxic concentrations (10 μM and 1 μM respectively) were evaluated by immunofluorescence microscopy on Jurkat cells. After 6 hours treatment with paclitaxel, strong modifications of the microtubule network (that form bundles) were observed (Fig. 3C). However, in the presence of 10 μM Tat, we did not observe bundles or any other modification of the microtubule network.

By disturbing microtubule functions, MDAs generally lead to a mitotic block of treated cells. Although study have implicated the transactivation effect of Tat on cyclin and other gene implicated in cell cycle progression, we examined if Tat induced a mitotic block that could be due in part to the tubulin polymerization of Tat. Following treatment, the percentage of cells in various phases was obtained by flow cytometry analysis after propidium iodide incorporation. After 20 hours treatment, 1 μM paclitaxel blocks almost 50 % of the cells in the G2/M phase (Fig. 3B, G2/M values). This strong blockage in G2/M with paclitaxel attests that we were in the appropriate time conditions to evaluate the effects of Tat on cell cycle regulation in these Jurkat cells. A longer incubation time (*i.e. *48 h) in the presence of paclitaxel leads to 90 % of cell death (data not shown). After 20 hours treatment, the percentage of the G2/M cells in the presence of 1 μM Tat HxB2 or 10 μM Tat Oyi were 28 % and 32 % respectively and similar to the 33 % observed in the untreated cells (Fig. 3B). In the presence of 10 μM Tat HxB2 or 10 μM Tat Eli the percentage of G2/M cells (22 % and 24 % respectively), were lower than the control cells (33 %). This decrease of the G2/M is not correlated with an increase in G1, and seems to be related to the strong cell death (30 %) rather than to a specific block in another cell cycle phase. In parallel, DAPI results confirmed that the percentages of metaphasic cells were similar between untreated cells and Tat-treated cells (1 %), in contrast to paclitaxel-treated cells that were blocked in metaphase (50 %). Thus, contrary to paclitaxel, the different Tat variants did not lead to a G2/M block before inducing apoptosis.

### Tat induces the release of cytochrome c from isolated mitochondria

**Figure 4:**
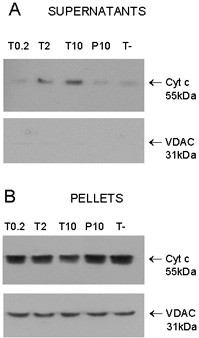
Tat induces cytochrome *c *release from isolated mitochondria. After 2 hour treatment with 10 μM (P10) of peptide 73–99 or 0.2 μM (T0.2), 2 μM (T2) and 10 μM (T10) of Tat Eli or without peptide (T-), samples were centrifuged and supernatants and pellets were separated. (**A**). Western blots of the supernatant with antibodies against cytochrome *c *(Cyt C) and with antibodies against VDAC. (**B**) Western blots of Mitochondria Pellets. Data are representative of four independent experiments.

MDAs induce apoptosis through the mitochondrial apoptosis pathway. Our previous work on paclitaxel showed that this agent is able to induce cytochrome *c *release from purified mitochondria [28]. To investigate whether Tat could directly target mitochondria, we isolated these organelles and incubated them with growing concentrations of Tat Eli (0.2 μM, 2 μM and 10 μM). Using Western Blot analysis, we detected cytochrome *c *in the supernatant of mitochondria incubated with Tat Eli (Fig. 4A, Cyt c). Enhanced amounts of cytochome *c *were observed with 2 μM and 10 μM Tat Eli (lane T10 and T2) whereas the level of cytochrome *c *with 0.2 μM of Tat Eli (lane T0.2) was comparable to the untreated mitochondria (lane T-). In parallel, cytochrome *c *levels decreased slightly in mitochondria treated with Tat, as revealed by Western Blot of the pellets (Fig. 4B, Cyt c). Levels of VDAC, mitochondrial outer membrane porin, were systematically measured to ensure that equal amounts of mitochondria were present in the pellets (Fig. 4B, VDAC), and that no mitochondria remained in the supernatants (Fig. 4A, VDAC).

## Discussion

Our *in vitro *study clearly shows that Tat directly interacts with microtubules and is able to enhance their formation. Turbidimetry tests showed that Tat strongly enhanced pure tubulin polymerization. The Tat concentration inducing tubulin polymerization is higher compared to the concentration of Tat in the plasma and it is possible that Tat concentration increases in the cell due to an active uptake similar to what is observed with paclitaxel. Furthermore, it is also possible that Tat concentration in the plasma is higher nearby HIV infected cells. We found that Tat acts qualitatively on microtubules making them shorter, such as paclitaxel, and also induces microtubule packing. These effects on microtubule formation are more pronounced than with paclitaxel as observed by turbidimetry and electron microscopy. The high effect of Tat on the self-association of tubulin is of interest for studies on the mechanism of microtubule formation and could be used in the design of new agents targeting microtubules.

We also showed that the full-length Tat protein is not necessary to obtain tubulin polymerization enhancement as peptides derived from its central region harbored the same properties as the full-length protein. These peptides contain the glutamine-rich region of Tat and confirms that the glutamine-rich region of Tat is involved in Tat mediated apoptosis [20]. It also contains the basic region (sequence 49–60) that is essential for Tat to cross membranes [36] and the adjacent region (sequence 38–48) that has been previously shown to be involved in the Tat-tubulin interaction [19]. In this study, we demonstrate that the N-terminus and the C-terminus of Tat are not necessary for the interaction with tubulin.

We showed that the more toxic Tat variants were also those that were more active in the polymerization of tubulin *in vitro*, suggesting that the toxicity of extra-cellular Tat is mediated by a mechanism involving microtubules. MDAs such as paclitaxel, which affect tubulin polymerization, generally disturb the microtubule network functions and inhibit cell proliferation. Contrary to paclitaxel, in the presence of high concentrations of Tat, we did not observe cell cycle arrest in G2/M. Moreover, by immunofluorescence microscopy, we did not detect any early modification of the microtubule network organization, even with high Tat concentrations. MDAs are able to induce apoptosis at lower doses than those required to induce bundles [21]. Interestingly, low concentrations of MDAs can suppress microtubule dynamics and disturb their functions, without modifying microtubule network morphology [21,27,37]. Thus, low cytoplasmic concentrations of Tat could modulate microtubule dynamics participating in the apoptotic signaling pathway induction in lymphocytes. The effect of Tat on microtubule dynamic needs to take in account that Tat could also modify the activity of proteins acting on microtubule dynamic such as LIS1 [38].

We hypothesize that Tat is able to induce the release of pro-apoptotic factors from mitochondria as we have previously shown this property for microtubule damaging agents [28]. We show here for the first time that Tat is able to directly affect isolated mitochondria and trigger cytochrome *c *release, a key event in the mitochondrial apoptotic pathway activation. This result is in agreement with those from Macho *et al. *that show that, in lymphocyte cultures under low serum conditions, Tat accumulates at the mitochondria. Moreover, this localization correlated with disruption of the mitochondrial membrane potential [39], a process that leads to the release of pro-apoptotic factors such as cytochrome *c*. Thus, we show in this study that secreted Tat can both act on tubulin polymerization and on the mitochondria *in vitro*.

Interestingly, although our study involves only three Tat variants, our results show that, in comparison with Tat Oyi, the Tat HxB2 and Tat Eli derived from RP are more potent in tubulin polymerization and apoptosis induction in T-cell. This open the possibility that these properties of Tat could be associated with more severe disease phenotype.

Apoptosis of bystander cells has been demonstrated to be very important in AIDS [40] and the rate of lymphocyte apoptosis has been correlated with progression rates [41]. It now appears that extra-cellular Tat secreted from HIV-infected cells is involved in the apoptosis of non-infected T cells. The elucidation of the mechanism responsible for Tat-mediated inhibition of the immune response therefore should have a tremendous impact for AIDS therapy.

## Methods

### Protein synthesis, Purification and Biochemical Characterization

Tat variants and their derived peptides were synthesized with an ABI 433A peptide synthesizer (Perkin Elmer, Applied Biosystem Inc.) with FASTMoc chemistry according to the method of Barany and Merrifield [42] on 4-hydroxymethyl-phenoxy-methyl-copolystyrene-1% divinylbenzene preloaded resin (HMP; 0.50–0.65 mmol; Perkin Elmer, Applied Biosystem Inc., Foster City, CA), as previously described [43,44]. Purity and integrity of proteins were confirmed by amino acid composition (6300 Beckman analyzer), partial sequence analyses (473A Protein Sequencer, Applied Biosystem) and by MALDI-TOF mass spectrometry (Perspective Biosystems, Voyager DE-RP).

### Purified Lamb Brain Tubulin

Lamb brain tubulin was purified by ammonium sulfate fractionation and ion-exchange chromatography, stored in liquid nitrogen and prepared for use as described [45,46]. During this purification particular caution was taken to remove microtubule-associated proteins (MAPs). Protein concentrations were determined spectrophotometrically with a Perkin-Elmer spectrophotometer Lambda 800. Tubulin extinction coefficient is ε_275 nm _= 1.07 L·g^-1^·cm^-1 ^in 0.5% SDS in neutral aqueous buffer or ε_275 nm _= 1.09 L·g^-1^·cm^-1 ^in 6 M guanidine hydrochloride.

### Tubulin polymerization

*In vitro *studies have been derived from experiments that show that the variation of temperature permits the disassembly/reassembly cycles of tubulin in the presence of GTP and Mg^2+ ^[47,48]. Microtubule assembly was performed in 20 mM sodium phosphate buffer, 1 mM EGTA, 10 mM MgCl_2_, and 3.4 M glycerol, pH 6.5. The various concentrations of Tat or paclitaxel were mixed with tubulin at 4°C. The reaction was started by warming the samples to 37°C in a 0.2 × 1 cm cells, and the mass of polymer formed was monitored by turbidimetry at 350 nm using a Beckman DU7400 spectrophotometer thermostated. Depolymerization occurs when the temperature is lowered to 10°C. The data showed in fig 1a were representative of three independent experiments. ΔOD_350 nm _values correspond to values without the buffer. The microtubule mass was determined by centrifugation assay. Samples were taken at the turbidity plateau at 37°C. They were ultra-centrifuged at 50,000 rpm for 15 min at 37°C using fixed angle rotor TLA 100.2 in a TL100 Beckman apparatus. In these conditions, microtubules precipitate in the pellet and non- polymerised tubulin remains in the supernatant. Pellet and supernatant were loaded separately on 10 % acrylamide SDS-PAGE, colored with Coomassie blue after migration (Fig. 1C).

### Electron microscopy

Small aliquots of tubulin associated with Tat were adsorbed on coated Formvar films on copper grids. They were negatively stained for 1 min in 2 % uranyl acetate and observed with a Jeol 1220 electron microscope.

### Cell Culture

The lymhoblastoid Jurkat cell line was cultured in RPMI 1640 supplemented with 2 mM ultraglutamine (BioWhittaker, Verviers, Belgium), penicillin (100 U/ml), streptomycin (100 μg/ml), and 10 % heat-inactivated fetal calf serum (BioWhittaker, Verviers, Belgium). Human neuroblastoma SK-N-SH cells were routinely maintained at 37°C and 5 % CO_2_, in standard culture RPMI-1640 medium (BioWhittaker, Verviers, Belgium) containing 10 % fetal bovine serum (BioWhittaker), 2 mM glutamine (BioWhittaker, Verviers, Belgium), 1 % penicillin and streptomycin (BioWhittaker, Verviers, Belgium). Exponentially growing cells (10^5 ^cells/ml) were seeded 3 days before paclitaxel or Tat treatment.

### Drugs and Antibodies

Stock solution of paclitaxel (Sigma) was prepared in DMSO. The final concentration of DMSO used in cell culture was less than 0.05 %. For Western blotting, anti-VDAC antibody (anti-porin, 31 HL France Biochem, Meudon, France) was used at 1/500 and anti- cytochrome *c *antibody (7 h82C12; PharMingen, San Diego, CA) was used at 1/1000. Secondary antibody was goat antimouse monoclonal antibody conjugated with peroxidase (Jackson ImmunoResearch Laboratories, USA). The antibodies used for immunofluorescence microscopy were anti-α-tubulin (clone DM1A Sigma, Saint Louis, USA) and FITC mouse anti-goat IgG (Jackson ImmunoResearch Laboratories, USA).

### Cell death analysis

Jurkat cells at a concentration of 5 × 10^5 ^cells/ml were incubated in the absence or presence of Tat or paclitaxel at different concentrations (see Fig. 3). After 20 hours incubation at 37°C, they were counted under optic microscope and cell death was estimated by trypan blue exclusion. Cells were then permeabilized with glacial alcohol, stained with propidium iodide and DNA content was measured by flow cytometry (Facs Calibur, Becton Dickinson, Mississauga, Canada). Cytogram analysis was performed with Cell Quest Pro^® ^software (Becton Dickinson, Mississauga, Canada). The proportion of hypodiploid cells was used as an estimate of apoptosis. Cell death was further analysed using fluorescence microscopy of cells stained with DAPI. 5 × 10^5^. Jurkat cells/ml were incubated at 37°C for 20 hours with various Tat or paclitaxel in Lab-Tek chamber slides. (Nalge Nunc International., USA). After centrifugation for 10 min at 3500 rpm, spin cells were fixed with 3.7 % formaldehyde, permeabilized with 0.1 % saponin and treated with PBS containing 10 μg/ml 4'6-diamidino-2-phenylindole (DAPI) (Sigma). The morphology of the cell nuclei was observed with a fluorescence microscope using an excitation wavelength of 350 nm. Nuclei were considered to have the normal phenotype when glowing bright and homogenously. Specific well drawn line up chromosome were seen in equatorial plates for cells in metaphase. Apoptotic nuclei were identified by the condensed chromatin gathering at the periphery of the nuclear membrane or a total fragmented morphology of nuclear bodies. Cells were counted and the percentage of normal., mitotic and apoptotic nuclei determined. Data indicated in the figure 3 are representative of three independent experiments. The apoptosis data obtained from the three independent experiments in the presence of paclitaxel and various Tat were compared using one-sided ANOVA following baseline subtraction using the values of untreated control cells.

### Indirect immunofluorescence staining of alpha-tubulin

10^6 ^Jurkat cells/ml were treated for 6 hours with 10 μM Tat HxB2 or 1 μM paclitaxel and were processed for tubulin indirect immunofluorescence staining as previously described [49]. After incubation with paclitaxel or Tat for 6 hr in lab-tek chamber slides (Nalge Nunc International, USA), the cells were centrifuged for 10 min at 3500 rpm. They were then fixed with 3.7 % formaldehyde and permeabilised with 0.1% saponin. Immunofluorescence microscopy of the microtubule network was performed using anti- α-tubulin antibody (Amersham, USA) and an FITC-conjugated secondary antibody (Jackson ImmunoResearch Laboratories, USA).

### Effect on mitochondria

Mitochondria were isolated by cell fractionation from the neuroblastoma SK-N-SH cell line as previously described [28]. 9 × 10^7 ^cells were suspended in a sucrose buffer (250 mM sucrose, 1 mM dithiothreitol, 10 mM KCl, 1 mM EDTA, 1 mM EGTA, 1.5 mM MgCl_2_, phenylmethylsulfonyl fluoride, protease inhibitors, 20 mM Hepes, pH 7.4) at 4°C, homogenized with 50 strokes in a glass homogenizer (Kimble Kontes, Vineland, NJ), and centrifuged twice at 800 g for 10 min. The supernatants were then centrifuged at 15,000 g for 10 min at 4°C. Mitochondrial pellets were immediately washed three times in the sucrose buffer. Isolated mitochondria were aliquoted and incubated for 2 hr at 37°C with various Tat concentrations. After centrifugation (15,000 g for 10 min at 4°C), pellets and supernatants were carefully separated. Mitochondrial pellets were lysed in lysis buffer (62.5 mM Tris.HCl; pH 6.8, 0.5% SDS, 5% mercaptoethanol, 10% glycerol) and loaded in parallel with the supernatants on a reducing 15 % SDS-PAGE gel. The gel was processed for immunoblotting and revealed using antibodies against VDAC and cytochrome c. Anti-VDAC antibody (Calbiochem, USA) was used to ensure that equal amounts of mitochondrial proteins were loaded on the gels and that no mitochondria or lyzed mitochondria were present in the supernatant. Secondary antibodies were added and visualization was carried out using an enhanced chemiluminescence detection kit (ECL, Amersham, Aylesbury, United Kingdom). The results presented are representative of four independent experiments.

## List of abbreviations

HIV, human immunodeficiency virus

MDA, microtubule damaging agent

VDAC, voltage-dependent anion selective channel.

## Competing interest

The author(s) declare that they have no competing interests.

## Authors' contributions

JdM carried out most of the experimental work, performed the experimental design and drafted the manuscript. MC performed part of the cell, mitochondrial and immunofluorescence study directed by DB. PB performed tubulin purification, part of tubulin experiments on polymerization, quantification of microtubules and electron microscopy directed by VP. GRC, SL, SO, DE and JW synthezised the peptides and characterized them biochemically and biophysically. CP performed FACS studies. EPL participated in the design of the study, the redaction of the manuscript and funded the studies.
